# Inhibitors of NLRP3 Inflammasome in Ischemic Heart Disease: Focus on Functional and Redox Aspects

**DOI:** 10.3390/antiox12071396

**Published:** 2023-07-07

**Authors:** Pasquale Pagliaro, Claudia Penna

**Affiliations:** 1 Department of Clinical and Biological Sciences, Turin University, Orbassano, 10043 Turin, Italy; claudia.penna@unito.it; 2 National Institute for Cardiovascular Research (INRC), 40126 Bologna, Italy

**Keywords:** inflammation, reactive oxygen species, ischemia/reperfusion injury, endothelial dysfunction

## Abstract

Myocardial ischemia-reperfusion injury (MIRI) is caused by several mechanisms, including the production of reactive oxygen species (ROS), altered cellular osmolarity, and inflammatory response. Calcium overload, altered oxygen levels, and mitochondrial ROS are also involved in these MIRI processes, resulting in the irreversible opening of the mitochondrial permeability transition pore (mPTP). These mechanisms and processes are associated with NLRP3 inflammasome priming and activation, which can also induce cell death by pyroptosis through the up-regulation of the caspase-1 pathway and IL-18 release. In addition, endothelial dysfunction, both in the presence and absence of MIRI, is also accompanied by altered oxygen levels, decreased nitric oxide production, and ROS overproduction, resulting in the expression of adhesion molecules and leukocyte infiltration in which the NLRP3 inflammasome plays a central role, thus contributing, through endothelial dysfunction, to the alteration of coronary flow, typical of ischemic heart disease. Given the intricate interrelationship between ROS and NLRP3, ROS inhibitors can reduce NLRP3 inflammasome activation, while NLRP3 inhibitors can reduce oxidative stress and inflammation. NLRP3 inhibitors have been intensively studied as anti-inflammatory agents in basic cardiovascular sciences. In this review, we analyze the interrelation between ROS and NLRP3 in ischemic heart disease and the effects of some NLRP3 inhibitors as possible therapeutic agents in this disease condition. All compounds considered in this review need larger studies to confirm their appropriate use in clinical scenarios as anti-ischemic drugs.

## 1. Introduction

Myocardial ischemia-reperfusion injury (MIRI) is the aggravation of myocardial damage caused by the rapid reperfusion of ischemic myocardium. In fact, reperfusion causes further damage to the ischemic tissue in the experimental and clinical scenarios of ischemic heart diseases (IHD). The reperfusion damage and subsequent cell death occur primarily as a consequence of massive reactive oxygen production (ROS) production, altered cellular osmolarity, and subsequent inflammatory response, including neutrophil infiltration. The inflammatory response not only affects the local aspect but is generalized and, in severe cases, can lead to a syndrome of multiorgan dysfunction [1]. Coronary circulation is both the culprit and victim of MIRI, which is caused by complex mechanisms that include intracellular, extracellular, and mechanical processes, all intertwined with inflammatory processes [2,3]. Among the inflammatory processes that are triggered by ROS but which in turn generate and exacerbate redox-related pathologies, researchers are paying particular attention to the activation of Nod-like receptor (NLR) proteins, particularly the NLR pyrin domain containing 3 (NLRP3) [4], which is the main and most characterized member of the NLR family [5].

Actually, NLRs are a protein family of intracellular sensors, whose members share a conserved central nucleotide bond, an oligomerization domain (NOD), and a variable N-terminal effector domain and are rich in leucine repeat (LRR) [6,7,8].

Therefore, the innate immune system intervenes in IHD through the recognition of receptor patterns (PRR) capable of detecting the presence of both pathogenic microbes and other endogenous or exogenous pathogens, such as damage-associated molecular patterns (DAMPs) or pathogens (PAMPs) [9].

The activation and regulation of the NLRP3 inflammasome is a complex two-step process consisting of priming and activation. Upon recognition of PAMP or DAMP, the inflammasome is primed. The first phase, or priming, is carried out through the activation of the nuclear factor kappa B (NF-κB) pathway, which leads to the up-regulation of NLRP3 and pro-IL-1β proteins and changes in the post-translational modifications (PTMs) of NLRP3, such as ubiquitination and phosphorylation, which empower NLRP3 and promote inflammasome assembly. After priming, the second phase, or activation phase, leads to conformational changes in NLRP3 and PTMs that enable NLRP3 oligomerization and consequently inflammasome activation. This phase is characterized by an up-regulation of the protein expression of some components of inflammasomes (NLRP3, apoptosis-associated speck-like protein (ASC), caspase-1, and pro-interleukin (IL-1β). Upstream, inflammasome activation is also regulated by various ion signals (K^+^, Ca^2+^, Cl^−^), mitochondrial dysfunction, and lysosome disruption [8,10].

### MIRI Activates Various Cell Death Pathways

Numerous cell death pathways, including but not limited to pyroptosis, apoptosis, necrosis, and autophagy, are present in MIRI [6,7]. An important mechanism of MIRI is sterile inflammation with subsequent *pyroptosis* cell death [7]. Therefore, NLRP3 perceives cardiomyocyte injury and enrolls an ASC (apoptosis-associated speck-like protein carrying a CARD (C-terminal caspase recruitment domain) and procaspase-1 to shape inflammasome complexes, which initiate the pyroptosis pathway. This involves gasdermin D (GSDMD) activation, which represents the classical pathway of pyroptosis cell death [11]. NLRP3 activation is the major player in this type of controlled cardiomyocyte cell death, which is characterized by the development of cell membrane holes, the release of pro-inflammatory cytokines, and cell lysis [8]. Specifically, pyroptosis is a mode of cell death by which the immune system responds to endogenous damage and pathogens. Indeed, pyroptosis has been defined as a sort of programmed GSDMD/NLRP3-dependent necrosis and is mediated by GSDMD cleavage by caspase-1 [12]. Apoptosis and pyroptosis are similar in that both involve DNA damage, a positive terminal deoxynucleotidyl TUNEL (transferase-mediated UTP nick end-labeling) result, and the presence of annexin-5. However, in contrast to apoptosis, pyroptosis damages the cell membrane quickly and irreparably [13]. Both pyroptosis and apoptosis exhibit chromatin condensation, even though the nucleus remains intact in apoptosis. Since in pyroptosis, a pivotal role is played by GSDMD, the deletion of the GSDMD gene prevents both pyroptosis and IL-1β secretion by macrophage cells [14].

Of note, functional and physiological *autophagy*, another regulated cell death, would have the overall effect of minimizing NLRP3 activation and reducing cytokine secretion. However, abnormalities in autophagy pathways may result in ineffective or incomplete autophagy and mitophagy, thereby inducing the activation of NLRP3 and exacerbating myocardial damage [12,15]. *Mitophagy* is also among the mechanisms activated by MIRI. Lemasters introduced the term mitophagy in 2005 to highlight a very particular autophagic process that affected the mitochondria [16]. Indeed, mitophagy is a process aimed at maintaining mitochondrial homeostasis at the cellular level, which causes the degradation of dysfunctional mitochondria [17].

Recently, *ferroptosis* has been reported among the cellular death modalities activated by MIRI [18]. Ferroptosis is an iron-dependent form of controlled cell death characterized by intracellular iron overload and redox system dysfunction, inducing lipid peroxidation. It is closely correlated with the induction of inflammatory signaling pathways, such as JAK-STAT, NF-κB, inflammasome, cyclic GMP–AMP synthase (cGAS)–stimulator of interferon genes (STING) pathway (cGAS-STING), and MAPK signaling pathways, and vice versa. As a matter of fact, the inflammatory response is a significant factor in challenges with iron metabolism and redox system dysfunction [19]. Additionally, mTOR is involved because its deletion causes an excessive amount of cell death, while high levels of mTOR block the cell death brought on by the inducer of ferroptosis, *erastin* [18]. Actually, the mechanism underlying ferroptosis is not fully understood, but it is unquestionably related to mitochondrial dysfunction [20]. Indeed, a reduction in mitochondrial volume, an increase in the density of the bilayer membrane, and a rupture of the outer mitochondrial membrane have all been observed, suggesting that the morphology and size of the mitochondria can be affected [21].

## 2. Mechanisms Involved in MIRI and Interactions with NLRP3

**Figure 1:**
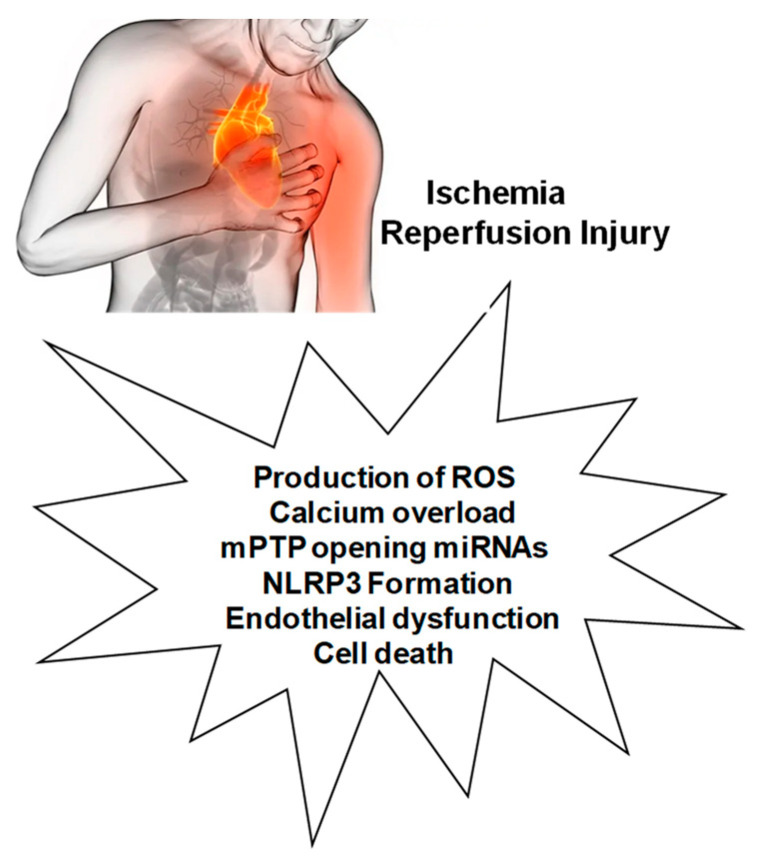
Factors involved in determining myocardial ischemia/reperfusion injury. Mitochondrial permeability transition pores, mPTP.

The main mechanisms involved in MIRI (Figure 1) and intertwined with NLRP3 include ROS production, calcium overload, mPTP opening, MiRNAs, and endothelial dysfunction.

### 2.1. Production of ROS

Following MIRI, a large amount of ROS is produced during the early stages of reperfusion. During ischemia and reperfusion, ROS are produced by different mechanisms at the mitochondrial level, but they can also be produced by the intervention of cellular enzymes, such as NADPH oxidases (NOX) family, cyclooxygenase (COX) and lipoxygenase (LOX) [22]. These ROS are capable of causing cellular damage by inducing lipid peroxidation, disrupting cell signaling pathways, and activating pro-inflammatory factors. The high production of ROS results in the opening of mitochondrial permeability transition pores (mPTP), leading to mitochondrial damage [17] and the induction of ROS-induced ROS release (RIRR). ROS during MIRI promotes tissue inflammation and the activation of the NLRP3 complex in various organs, including the heart. In addition, it appears that NLRP3 itself directly or indirectly induces ROS production at the mitochondrial level [23]. ROS are able to induce the synthesis of cytokines, including IL-18, inducing the inflammatory state of tissues and promoting both apoptosis and calcium overload. Thus, the high amount of ROS results in cell death, including apoptosis and pyroptosis. ROS production also appears to be induced by its ability to induce the release of inflammatory factors. Thus, ROS are involved in a number of early and late self-ingravescent mechanisms. Despite the well-known deleterious effects, low levels of ROS have an essential, albeit double-edged, role in cardioprotection [24,25,26]. Similarly, a double-edged role has been reported for NLRP3 [27,28]. This double-edged role for NLRP3 is shown in animal model NLRP3 knockout (KO) where the cardioprotective effects of ischemic preconditioning are reduced and the levels of signal transducers and activators of transcription 3 (STAT3) proteins are decreased in this animal model [27,28]. ASC, another component associated with NLRP3, appears to have a protective role, although the number of studies is currently very small. In a mouse model of ASC−/−, no action on ischemic preconditioning-induced protection was demonstrated for this factor. Indeed, ASC deficiency had no effect on an ischemic preconditioning protocol [28]. However, in another work, it was reported that either the absence of NLRP3 or of ASC determined the loss of protection [29]. Obviously, further studies are necessary to clarify these discrepancies.

### 2.2. Calcium Overload

As a result of MIRI, a disordered calcium distribution is established, leading to dysfunctional calcium homeostasis, and an anomalous increase in intracellular calcium concentration, also referred to as calcium overload, develops. This overload also affects the mitochondrial level and is accompanied by both a decrease in mitochondrial membrane potential and ATP content and mPTP opening. A close correlation between calcium overload/NLRP3 expression/pyrotosis has been demonstrated in several tissues, including the heart [11,30]. Calcium overload in MIRI operates as an excitatory element of oxidative stress, which in turn induces inflammasome formation [11]. Notably, Mo et al., in a hypoxia/reperfusion (H/R) model of adult rat cardiomyocytes, demonstrated that calcium overload can induce pyroptosis by up-regulating the NLRP3/caspase-1 pathway [31].

### 2.3. Role of mPTP Opening

Mitochondria are the central nodes of the cell and are in charge of cellular energy production through oxidative phosphorylation (OXPHOS) [32]. These organelles are influenced negatively by both low oxygen levels following ischemia and, as seen previously, by ROS produced during the early stages of reperfusion. Both of these factors participate in cell death due to the irreversible opening of the mPTP [33]. The opening of the mPTP leads to a loss of mitochondrial membrane potential (ΔΨm), a loss of OXPHOS activity, and a subsequent drastic reduction of ATP [34]. This leads to mitochondrial osmotic shock and the rupture of the outer mitochondrial membrane and, consequently, to all forms of cardiomyocyte death, including pyroptosis [7,35].

### 2.4. Endothelial Dysfunction

Endothelial dysfunction involves the decreased production of nitric oxide (NO), expression of adhesion molecules, the adhesion of leukocytes to the endothelium, and the infiltration of leukocytes. All these phenomena are mechanisms involved in MIRI, leading to the so-called “no-reflow phenomenon”, which is especially due to limited NO availability, resulting in vasoconstriction and micro-thrombus formation in the lumen of small vessels [3,36,37]. In endothelial dysfunction, the activation of NF-κB and other transcription factors, with increased expression of cell adhesion molecules, play a pivotal role in inducing inflammatory processes [3,36]. Of note, NF-κB is central to NLRP3 priming and activation [4,38].

Another harmful aspect induced by early reperfusion is inflammatory damage mediated by the activation of mast cells and neutrophils, whose products act as chemoattractants for other leukocytes [39]. Recently cathepsins, proteases involved in multiple pathophysiological roles, have attracted the attention of researchers. In particular, cathepsin G, a modulator of neutrophil chemoattractant, induces morphological modifications that break focal adhesion and intracellular contacts with cardiomyocytes and is involved in MIRI [39,40]. Indeed, cathepsin G inhibition with DCCI (dual inhibitor of cathepsin G and chymase) limits MIRI and damages [41].

## 3. Role for microRNAs in MIRI and NLRP3 Activation

MicroRNAs, also known as miRNAs or miRs, are single-stranded noncoding RNAs, 21–25 nucleotides long and involved in the post-transcriptional regulation of numerous genes. They are now being recognized as crucial components of extracellular vesicles as well as significant contributors to the exacerbation and amelioration of MIRI, activation of NLRP3, and other processes [42]. Several microRNAs, including miR-34a-p, miR-363, miR-449a, miR-92a, and miR-155, act in favor of MIRI development and heighten damage in various ways. Increased ROS production, apoptosis, and macrophage activation are some of the potential pathways [43]. A few microRNAs have been identified as having the ability to lower MIRI, including miR-146a and miR-210, both of which are capable of increasing ischemic tissue angiogenesis [43]. A number of microRNAs, like miR-155, miR-146a, miR-21, and miR-132, were also connected to TLR4/MyD88/NF-kB signaling and mediated the two steps of NLRP3 activation. In particular, the second step of NLRP3 activation is mediated by miR-223 [44,45]. Therefore, by inhibiting the aforementioned microRNAs, NLRP3 activation and the release of IL-1β and IL-18 could be prevented. Moreover, many of the aforementioned microRNAs, particularly miR-155 and miR-146a, are crucial for the development of atherosclerosis [46]. Indeed, the anti-inflammatory properties of miR-146a counteract those of miR-155. By inhibiting IL-6, IL-1, IL-8, and TNFα, miR-146a acts as an important anti-inflammatory and atheroprotective agent, acting as a “brake” on inflammation. Through targeting TLR4, it prevents oxidized LDL-induced lipid accumulation and inflammatory response. This is an important role for miR-146a in the inflammatory process associated with thrombosis, namely thrombo-inflammation. Since miR-146a inhibits apoptosis, the inflammatory response, and fibrosis, its increase after a MI is cardioprotective. In contrast, low levels of miR-146a expression promote an expansion of the MIRI, apoptosis, and infarct size after acute coronary syndrome. Additionally, miR-21, miR-132, and miR-223 contribute to post-MIRI and damage. Recent studies on the role of microRNAs and inflammation-priming have led to the hypothesis that non-cardiovascular pathologies like systemic lupus erythematosus and rheumatoid arthritis may be related to altered miRNA/inflammasome expression [44].

## 4. Inflammasome and Redox Signaling

As mentioned above, various studies suggested that ROS are key factors contributing to the activation of the NLRP3 inflammasome. Yet, most of the NLRP3 inflammasome activators can lead to the production of ROS at the mitochondrial level in different cellular models. The mechanisms by which the NLRP3 inflammasome interacts and cross-talks with ROS production are not yet well understood. Both NLRP3 and ASC in physiological conditions are localized at the cytoplasmic level and in the endoplasmic reticulum. After activation, the two molecules translocate at the dynamic level platforms in mitochondria and endoplasmic reticulum (ER) contact (the so-called mitochondria-ER-associated membranes; MAM) [47]. This translocation could probably be due to the fact that the ROS involved are of mitochondrial origin. Nevertheless, following the accumulation of ROS there is the production of IL-1β at the cellular level, which supports the viewpoint that the activation of NLRP3 is ROS-dependent [10]. Important components of mitochondria are the various mitochondrial outer membrane channels located at the level of MAMs; these include voltage-dependent anion channels (VDACs), which play a central role in Ca^2+^ exchange, mitochondrial ROS production, and activation of the NLRP3 complex. Recently, it has been observed that blocking VDACs reduces both ROS production and NLRP3 inflammasome activation and IL-1β production. The close link between VDACs and the inflammasome has also been seen with the oligomerization of the channel itself causing NLRP3 activation and through the use of activators of the inflammasome complex that result in both channel oligomerization and mPTP opening [10].

The molecules most involved in the link between ROS and activation of NLRP3 are thioredoxin (TXNIP), NF-κB, and erythroid nuclear factor-binding transcription factor 2 (Nrf2) [48]. When subjected to stress, the ER produces ROS from NOXs. These enzymes can produce high quantities of ROS and can cause mitochondrial calcium disorders with a consequent loss of mitochondrial function, mPTP opening, and RIRR. In addition, NOX, particularly NOX4, is responsible for the activation of NF-κB through the production of ROS and consequently facilitates the initiation and activation of NLRP3 and the release of inflammatory cytokines [49]. It has been suggested that the component of the NLRP3 complex, NIMA-related kinase 7 (NEK7) may be the true sensor of ROS [50]. Conversely, *imiquimod*, an agonist of the toll-like receptor (TLR) 7 component, induces NLRP3 inflammasome activation in a ROS-dependent manner, even though cells lacking NEK7 fail to produce IL-1β in response to imiquimod stimulation [51]. It has also been reported that ROS play a central role in the priming step required to induce NLRP3 expression [51]. Indeed, the antioxidant NAC is able to inhibit caspase-1 activation in response to various NLRP3 stimuli (e.g., LPS/ATP, LPS/nigericin, or LPS/silica) [52]. Moreover, NLRP3 activation by fatty acids found in a high-fat diet (HFD) causes NLRP3 activation via the AMPK/autophagy/ROS pathway. In this model, inhibitors of ROS synthesis are able to reduce the activation of the NLRP3 inflammasome [51]. Finally, at the endothelial level, ROS can be defined as the point of contact between NLRP3 and endothelial dysfunction. Another point of contact between ROS and NLRP3 has been recently reported in LPS-induced sepsis, using a specific inhibitor for aquaporin 9 (AQP9). Indeed, the administration of the specific AQP9 inhibitor protects animals from sepsis-induced multiorgan dysfunction. This protection is attributed to a reduction in NLRP3 complex activation by oxidative stress, in particular, AQP9 blockade reduces H_2_O_2_ permeability in vitro [53].

**Figure 2:**
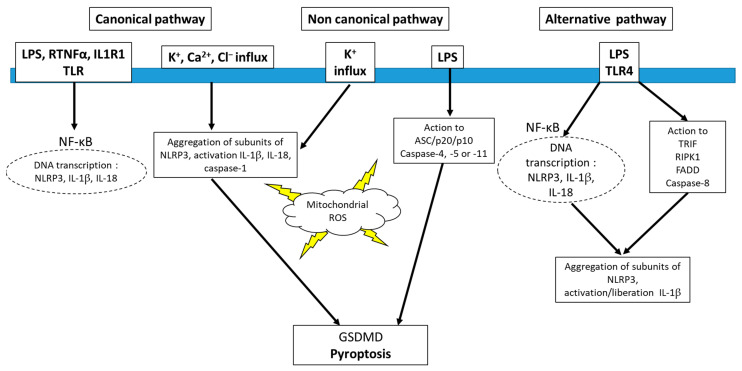
Schematic pathways for NLRP3 activation. Lipopolysaccharide, LPS; tumor necrotic factor receptor α, RTNFα; pyroptosis-inducing protein, gasdermin D, GSDMD; adaptor protein for TLR3, TRIF; FAS-associated death domain, FADD; receptor-interacting serine/threonine protein kinase, RIPK.

Recent studies have highlighted a non-classical pathway for NLRP3 activation. This pathway, referred to as a *non-canonical pathway*, seems to be caused by the involvement of caspases 4/5/11 autocleavage [54,55] (Figure 2). The active forms of these kinases induce pyroptosis with ATP release, pannexin-1 activation, and potassium ion entry, which in turn determines the activation of the NLRP3 complex and the release of cytokines [54,56]. Another activation of NLRP3 is indicated as “*alternative pathway*”. In this alternative pathway, attributed to LPS effects, TLR ligands enable IL-1β secretion without pyroptosis via TLR4-TRIF-RIPK1-FADD-CASP8 signaling upstream of NLRP3 [57]. ROS may be involved in all forms of NLRP3 activation. Indeed, ROS are shown to be among the first intermediate products induced by numerous NLRP3 inflammasome activators and are involved in the various mechanisms involved in both NLRP3 formation and activation [58].

## 5. Inhibitors of NLRP3

Given the central role of NLRP3 activation in IHD, several drugs have been investigated as anti-inflammatory agents in the cardiovascular field; some have failed and been discontinued (atreleuton, canakinumab, darapladib, losmapimod, methotrexate, PF-04191834, setileuton, varespladib, and veliflapon), while others are still in development (e.g., allopurinol, anakinra, colchicine, everolimus, IFM-2427, inzomelid, montelukast, somalix, tocilizumab, and ziltivekimab). Several compounds tested as NLRP3 inhibitors in the IHD scenario have been recently reviewed by Toldo et al. [15]. Here, we briefly consider only NLRP3 inhibitors and downstream interleukins, emphasizing redox aspects within the context of IHD, if possible. When not possible, we still mention their redox aspects in other fields to allow readers possible future consideration of these aspects in IHD as well.

*Colchicine* is an old anti-inflammatory drug, which recently was found to inhibit the assembly and activation of NLRP3. New knowledge about the mechanism of action has paved the way for investigations in the cardiology fields [59,60]. In a mouse model of non-reperfused acute myocardial ischemia (AMI), a high dose significantly inhibited the increase in inflammasome activity at 24 h after AMI [61].

Recently, colchicine was shown to significantly reduce IL-1β and ROS release in ATP-pretreated macrophage models [61].

*Glyburide/glibenclamide* has been demonstrated to inhibit IL-1β release in vitro. Although, the administration of glyburide inhibited NLRP3 inflammasome, at moderate doses would cause lethal hypoglycemia in mice [62,63]. A derivative of glyburide, 4-[2-(5-chloro-2-methoxybenzamido)ethyl]benzenesulfonamide, which also lacks the part responsible for inducing insulin secretion, was developed; this maintained the inhibitory effect against inflammasome and abolished the hypoglycemic effect of glyburide [64]. Reperfusion containing glyburide analogs reduced macromolecular aggregation of ASC and caspase-1 activity. This was accompanied by a reduction in the plasma levels of cardiac troponin I and infarct size [64].

In a mouse model of Parkinson’s disease in which inflammasome involvement has been demonstrated in previous studies [65], glibenclamide has recently been shown to reduce inflammasome levels and the release of related cytokines, by reducing the molecular activation of NF-κB and the expression/activation of NOX2 and inducible NOSynthase [66]. This is particularly relevant from the redox point of view, as it is known that both inducible NOS (iNOS) and NOX2 are the main enzymes responsible for ROS production in activated microglia and play an important role in controlling both microglial activation and oxidative stress [67].

*MCC950/CP-456,773* is a potent NLRP3 inhibitor [68,69]; it has been shown to be effective in a mouse model of encephalomyelitis with a constitutively active NLRP3 mutant. Treatment for about one month with MCC950 successfully rescued these mice [70]. Directly attaching to the NLRP3 Walker B site in the NACHT domain, MCC950 prevents NLRP3 from hydrolyzing ATP and closes the active conformation of the protein [57]. This inhibitor is able to affect all three NLRP3 activation pathways through mechanisms that are currently not fully understood [54]. It is known that following sepsis, the survivors show serious neurological damage [71], it has been observed that in a mouse model of sepsis induced by cecal ligation and perforation, the administration of MCC950 is able to reduce the oxidative damage at the level of proteins and lipids, maintaining the activity of superoxide dismutase (SOD) at the level of the hippocampus [71].

*IFM-2427* is one of the NLRP3 inhibitors being studied. Similar to MCC950, it contains a sulfonamide, but the urea and tricyclic part in MCC950 is modified with an acetamide linked to a substituted phenyl. On the opposite side of the molecule, the pyrrolic ring is exchanged for a diverse variety of aromatic heterocycles with a high preference for thiophenes and thiazoles. A phase I clinical trial exploring the effects of IFM-2427 in gout, Crohn’s disease, and coronary artery disease was initiated in 2019 [72]. This inhibitor may be useful in preventing atherosclerosis, in which redox aspects play a pivotal role [73].

*Inzomelid* and *somalix* are two NLRP3 inhibitors recently developed by inflazome (now acquired by Roche). Twenty-four patent applications for both compounds were published in 2019, and in all patents were compared to the sulfonylureas series of MCC950. Both compounds are in clinical trials. In particular, somalix is being studied as an oral drug against inflammatory conditions such as arthritis and cardiovascular disease [68]. These two inhibitors may also be useful in the prevention of atherosclerosis, in which redox aspects play a central role [73].

*INF4E* is an acrylamide derivative developed by Bertinaria and Coll [74]. This class of drugs is considered a covalent inhibitor of NLRP3 and its ATPase activity, which is normally required for inflammasome activation. Infusion of INF4E before ischemia, in an ex vivo model of MIRI, displayed a significant increase in left-ventricle-developed pressure 60 min after ischemia and a significant reduction of infarct size [75].

INF4E influences the expression of nuclear factor-E2-related factor (NRF) 2 but not that of NRF1 in post-ischemic myocardium [74]. A similar compound to INF4E is INF39 [76].

*BAY 11-7082*, an NF-κB inhibitor, has been shown to directly suppress NLRP3 ATPase activity. In a rat model of MIRI, pretreatment with BAY 11-7082 significantly reduced infarct area and cardiac fibrosis and improved left ventricular fractional shortening [77]. In a recent study by Kleniewska et al. [78], it has been reported that the administration of BAY 11-7082 significantly reduced both H_2_O_2_ and thiobarbituric-acid-reactive substances (TBARS) production, thus suggesting a less production of ROS in a model of hepatic dysfunction induced by exogenous endothelin-1 (ET-1)-infusion [78]. This antioxidant effect by BAY 11-7082 was also observed in human endothelial cells exposed to resistin, an adipokine considered an inflammatory marker in atherosclerosis [79] showed that this antioxidant effect by BAY 11-7082 also occurs in Kupffer cells, where the inhibitor causes a reduction in ROS production and increases reduced glutathione (GSH) levels.

*Curcumin* is a polyphenolic compound of the rhizomes of turmeric. This compound displays a beneficial role in various inflammatory diseases, in particular, diabetes and cardiovascular diseases It exerts its effect by inhibiting NF-κB, extracellularly regulated protein kinases (ERK) 1/2 and c-Jun N-terminal kinase (JNK), reducing the production of ROS, limiting the entry of potassium and blocking the action of ASC [57].

*OLT1177/dapansutrile* is an active β-sulfonylnitrile molecule that showed safety when given orally in humans. It inhibits NLRP3’s ATPase activity and hinders the interaction of NLRP3 with ASC and caspase-1 [57]. It was tested in mice models of 30-min coronary artery ligation followed by OLT1177 administration. It displayed a significant reduction in the infarct area and plasma troponin I, as well as preservation of LV fractional shortening. Of note, Marchetti et al. [80] reported an inhibitory effect on NLRP3 ATPase activity, resulting in decreased NLRP-ASC oligomerization; however, no effects on receptor—absent in melanoma-2 (AIM2) or NLR CARD domain containing 4 (NLRC4) inflammasomes were observed.

*1,2,4-Triazine derivatives* block the action of IL-18, IL-1α, and IL-1β in cardiovascular disease. The expression and plasma levels of these ILs increased after reperfusion in several animal models of myocardial ischemia. The role of IL-18 was studied in MIRI in an experimental heart transplantation model. Treatment with an antibody neutralizing IL-18, 1 h before ischemia/reperfusion, significantly reduced the infarcted area [81]. Gu et al. [82] explored the cardioprotective effects of treatment with GSK1070806a, a monoclonal antibody against IL-18, which was also tested in patients with inflammatory bowel disease or diabetes mellitus [83,84]. In further studies in mice after acute infarction, the selective inhibition of IL-1β was investigated using an IgG2a monoclonal antibody directed against IL-1β, a murine analog of canakinumab, a human monoclonal antibody that blocks IL-1β [85]. Finally, anakinra, a widely studied recombinant IL-1 receptor antagonist, blunted the adverse remodeling process and the rate of myocardial apoptosis and improved left ventricular contractile function after acute infarction. In a porcine model of coronary occlusion and reperfusion, the infusion of anakinra for 15 min before ischemia for 75 min and treatment once a day after ischemia/reperfusion preserved left ventricular ejection fraction, decreased IL-1β levels in the myocardium, reduced infarct size, and attenuated neutrophil influx into the myocardium, confirming the efficacy of the treatment in the large animal model [86]. Anakinra also possesses antioxidant activity due to increased expression of mitochondrial SOD2. Specifically, from the results reported by He et al. [87], it appears that anakinra prevents SOD2 degradation by interfering with the USP36-COPS3 complex rather than by inducing its expression. Of note, anakinra is approved by European and U.S. pharmaceutical agencies for the treatment of rheumatoid arthritis and has been included in clinical trials against MIRI (https://clinicaltrials.gov/ct2/show/NCT01950299, accessed on 6 June 2023). Completed pilot clinical trials on the use of anakinra in patients with reperfused STEMI undergoing primary percutaneous coronary intervention showed that IL-1 blockade was safe and reduced C-reactive protein (CRP) levels at 72 h and the rate of new-onset cardiac failure after STEMI infarction [88,89,90]. In the MRC-ILA phase II clinical cardiac trial with anakinra used in non-STEMI coronaropathy, a similar reduction in acute inflammatory response was observed [91]. At 30 days and 3 months, there were no changes in adverse clinical events, but at 12 months, ischemic events had decreased in anakinra-treated patients compared with placebo-treated patients. Phase II clinical trials with anakinra used in patients with heart failure also appear to confirm the beneficial role of IL-1 inhibition [86,92].

All the compounds considered in the above paragraphs require larger trials to confirm their appropriate use in clinical scenarios.

Table 1 provides an overview of the NLRP3 inflammasome’s “priming” and “activating” mechanisms, NLRP3 pathway targets, and potential inhibitors.

## 6. Conclusions and Perspectives

The results reported in the present review support the importance of the NLRP3 inflammasome and its associated cytokines in a number of cardiac and vascular diseases as well as in several cell death modalities. Indeed, in the development and pathogenesis of various acute and chronic cardiovascular diseases, the action of the NLRP3 inflammasome, the subsequent production of IL-1β and IL-18, and cell death by pyroptosis are proving to be major players. Clinical evidence and experimental models suggest that NLRP3 inhibition has the potential to be a successful strategy for reducing heart damage, improving myocardial function, and avoiding unfavorable remodeling. Recently, several more or less specific inflammasome inhibitors have been developed to test the hypothesis that reducing NLRP3 inflammasome levels may improve clinical outcomes [93,94]. In particular, with some NLRP3 inhibitors like colchicine, OLT1177, and INF4E, encouraging outcomes have been seen, pointing to the availability and efficacy of this strategy. Early clinical trials using already approved inhibitors show promise in limiting the effects of the pathogenic NLRP3 inflammasome in cardiovascular disorders [15,93,94]. Moreover, additional NLRP3-specific inhibitors are being developed. For instance, specific treatments targeting the NLRP3 inflammasome have shown promise in reducing infarct size and preventing heart failure after acute myocardial infarction. The activation of survival pathways and an improvement in mitochondrial function have been linked to the cardioprotective effects of NLRP3 inhibitors, including INF4E, which improve cardiac function and postischemic outcomes [9]. Very recently, a novel NLRP3 inhibitor (1,3,4-oxadiazol-2-one derivative 5; INF200) has been tested as a pharmacological agent to mitigate cardiac and metabolic complications in an experimental model of diet-induced metaflammation [95].

It is important to note that NLRP3 priming and activation are largely influenced by redox stress. For example, NF-kB is involved in NLRP3 activation and oxidative stress response. Therefore, NLRP3 inflammasome can be deactivated by inhibitors of ROS production, and NLRP3 inhibitors can defuse oxidative stress. Indeed, there is an intricate interrelationship between ROS and NLRP3 that deserves to be studied in depth because it may be the key to breaking the *vicious* cycle between oxidative stress and inflammation (ROS begets NLRP3 and vice versa NLRP3 begets ROS). This *vicious* cycle is present in ischemic heart disease and many other cardiovascular diseases, including diabetes. In clinical studies published so far [94], the direction seems to be right, given the promising results but still awaiting validation in larger studies. To fully comprehend the precise mechanism of action and long-term effectiveness of NLRP3 inhibitors, particularly in the context of acute myocardial infarction, additional thorough analyses are required. For instance, as stated by Dekker et al. [96], acting before the significant conformational change required for ADP-ATP swap and subsequent inflammasome activation could be a more comprehensive strategy to limit the potential adverse effects. Therefore, we believe that further analysis regarding the protein structure of NLRP3 is essential in order to develop highly specific inhibitors able to block the complex in its folded (inactive) conformation. Further, we believe that determining the effectiveness of novel potential NLRP3 inhibitors directly during the reperfusion phase in experimental models of MIRI, including ongoing clinical trials [15,93,94], and assessing their beneficial effect over an extended period of time could be an effective way to set future experiments, particularly for the clinical transferability purpose.

## Figures and Tables

**Table 1:** “Priming” and “Activating” mechanisms for the NLRP3 inflammasome, potential inhibitors, and links between mechanisms and inhibitor effects.

Inhibitors	Links	Mechanisms	Refs.
**Priming**			
Curcumin	NF-κB	Reduce K^+^ flow, blocking the of ASC	[57]
Bay 11-7082	IKK-β	IKK-β induces phosphorylation and degradation of IκB proteins. IκB bind to NF-κB and prevent its translocation to the nucleus. The phosphorylation leads to the ubiquitination and proteasomal degradation of IκB, allowing NF-κB to be released and translocate to the nucleus.	[77,78,79]
**Activating Mechanism**			
Colchicine	ATP-gated cation channels (P2X2/P2X7); Lysosome; ASC	P2X2/P2X7 receptor binds extracellular ATP with opening of the channel (K^+^ channel); damage to lysosome induce K^+^ outflow; ASC polymers bind to pro-caspase-1 causing its polymerization. The pro-caspase polymers will be activated by the structure itself that has formed; the caspase-1 produced will activate the subsequent components (IL-1β and IL-18) and form the GSDMD pores responsible for pyroptosis.	[59,60,61,62]
INF4E, OLT1177, MCC950 and Bay 11-7082	ATPase domain, NACHT domain	The specific function of ATP hydrolysis is yet unknown, it allows the subsequent interaction between NLRP3 and ASC. Reduce redox damage, increase SOD activity	[57,70,71]
Glibenclamide/ Glyburide	NF-κB, NLRP3 oligomerization	Reduce the activation of NF-κB, expression/activation of NOX2 and iNOS, block the ADP-ATP switch in order to generate an active multimeric structure.	[66,67,68]
1,2,4-Triazine Derivatives.		Blockade of IL-18, IL-1α and IL-1β; prevents SOD2 degradation;	[82,83,84,85]

Table abbreviations: apoptosis-associated speck-like protein (ASC); gasdermin-D (GSDMD); inducible nitric oxide synthase (iNOS); inhibitor of nuclear factor kappa-B kinase subunit beta (IKK-β); nuclear factor kappa B (NF-κB); superoxide dismutase (SOD).

## Data Availability

Not applicable.
